# Small crystals, fast dynamics and noisy data are indeed beautiful

**DOI:** 10.1107/S2052252517009472

**Published:** 2017-06-30

**Authors:** Keith Moffat

**Affiliations:** a Department of Biochemistry and Molecular Biology, Institute for Biophysical Dynamics and Center for Advanced Radiation Sources, University of Chicago, 929 East 57th Street, Chicago, Illinois 60637, USA

**Keywords:** X-ray lasers, XFELs, biology, structure, dynamics

## Abstract

Structural biology at hard X-ray free-electron lasers is evolving rapidly beyond the experimental styles long established at storage ring sources. The comprehensive review by John Spence [*IUCrJ* (2017), **4**, 322–339] provides magisterial oversight of recent developments.

Free electron lasers represent a rare example of disruptive innovation in technology (Bower & Christensen, 1995 ▸). Such an innovation requires us to identify the ways in which the new technology differs significantly from the old and to decide how best to deploy that technology. Most importantly, we must consider what hitherto inaccessible scientific problems can now be successfully attacked, and reconsider what older problems can be better attacked. Free electron lasers had been developed at much longer wavelengths in the electromagnetic spectrum and used effectively for some time. It was however not a foregone conclusion that the self-amplified spontaneous emission process that underpins lasing would continue to hold for a hard X-ray FEL (XFEL), and that the roughly 100 m long undulator necessary for saturation of this lasing process could be constructed with the required precision. Even if lasing were achieved, it was not at all clear – particularly to reviewers ten years ago of grant applications to NIH in XFEL-based structural biology – that the prodigious peak brilliance of hard X-ray pulses could be harnessed to conduct any useful scattering or spectroscopic experiments before all atoms became fully ionized and the sample was destroyed.

We know how that all turned out: the world’s first hard X-ray laser, the Linac Coherent Light Source at the Stanford Linear Accelerator Center, speedily achieved lasing and was a roaring success. More to the point for X-ray experimentalists, a brief time window in the femtosecond range turned out to exist – as predicted by a few far-sighted scientists – during which scattering or spectroscopy data could be collected before the sample was indeed destroyed in the ultimate destructive experiment: ‘diffraction before destruction’ (Chapman *et al.*, 2011 ▸). As one of the pioneers of XFEL science in structural biology and its associated technologies, John Spence now presents a magisterial review (Spence, 2017 ▸) that illustrates his command of a large swath of the recent literature and of course, of the experiments that underlie it. The field is expanding so rapidly that this may turn out to be the last review that realistically covers the entire field. That expansion also illustrates that the field is quite immature and to many of us, wildly exciting. Developments are being vigorously pursued in such areas as the source itself (*e.g*. seeding; control of the bandpass of the X-ray spectrum or of the X-ray pulse structure), the X-ray optics (*e.g*. split-and-delay schemes to manipulate the X-ray pulse structure delivered to the sample; chirping of the pulse in time or space), the design of experiments (*e.g*. fixed target or jet-based modes of sample delivery; sample manipulation in dynamic experiments by rapid mixing, an intense pump pulse by a visible or IR laser, a temperature jump or an electric field jump; the combination of X-ray scattering and spectroscopic experiments), the detector and perhaps above all, in modes of analysis of the data. All are dealt with in the review, some at length, others more briefly.

Let’s consider modes of analysis. Scientific experiments generally aim at both accuracy (truthful measurements of the quantities of interest; no alternative facts accepted here!) and precision (the least uncertainty for a defined number of measurements or amount of precious sample). But free electron lasers are fundamentally based on the amplification of noise in the spatial distribution of electrons within an electron bunch as it traverses the long undulator. As a consequence the X-ray pulses emitted by an XFEL are extremely noisy, ‘spiky’ in both X-ray energy spectrum and time, with no correlation in spikiness from pulse to pulse: the epitome of irreproducibility. An individual XFEL pulse has a duration of 200 fs (adjustable from experiment to experiment), made up of spikes varying between near-zero and maximum intensity, where the width of each spike is a few fs. This irreproducibility is in complete contrast with the properties of the X-ray pulses from storage ring sources, which have been deliberately developed over the last 30 years or so to be remarkably stable in intensity, position and angle and to offer a smooth, near-Gaussian temporal profile of around 100 ps duration. The overall pulse length generally limits the time resolution of dynamic experiments at storage rings to at best several tens of ps and more often, around 100 ps (*e.g.* Pande *et al.*, 2016 ▸). Accurate measurements are greatly aided by any stable source, and high precision can generally be obtained with minimum amounts of sample. It looks as though without exception, ‘noise is bad for you’. In a remarkably novel and apparently quite general analysis of a time series of data, Ourmazd and colleagues (Fung *et al.*, 2016 ▸) demonstrate that in at least one class of a dynamic XFEL experiment, the time resolution is set by the spike width, not – as previously thought – by the XFEL pulse duration or timing jitter. Noise can be exceptionally good for you!

How general is this observation? If we’re creative enough, can noise be harnessed in other styles of dynamic experiments at XFELs, or even deliberately added by, for example, introducing modest spikes into the otherwise smooth temporal profiles of storage ring pulses? That remains to be seen. It is a fact that most of structural biology depends on the accurate measurement of small differences in X-ray scattering. Crystallographic examples include variation in structural amplitudes in *de novo* phase determination by anomalous scattering, in experiments with and without a ligand, or as a function of time in a dynamic experiment. There are parallel examples in solution scattering by SAXS/WAXS. We can now re-think ways in which desired accuracy can be achieved, while retaining the goal of using the least amount of valuable biological sample. As Spence notes, there is already extensive interplay between XFEL and storage ring experiments. Naturally enough, the earliest XFEL experiments were directly based on those conducted for decades at storage ring sources, though taking advantage of the enormous gains in peak brilliance to examine both much smaller samples and structural dynamics in the fs range. Conversely, the success of serial fs crystallography on micro- and nanocrystals at an XFEL has led to exploration of serial crystallography at storage rings, though on somewhat larger crystals and with much longer exposures. How the balance of experimental styles between storage rings and FELs will evolve remains to be seen. So does the question of the balance between single-particle structure determination using XFELs and advanced cryoelectron microscopy (Subramaniam *et al.*, 2016 ▸) .

Spence’s review, like any excellent review of a developing field, raises as many provocative questions as it answers. One of my own favorites is the time course and extent of X-ray-induced primary radiation damage in biological samples. It is sometimes claimed that a major advantage of FEL sources is that they enable damage-free X-ray data to be obtained. Not so! Certainly damage-reduced (the pulses in the fs range enable all secondary radiation damage to be outrun), but definitely not damage-free. Indeed as Spence emphasizes, for every hard X-ray photon that is elastically scattered and generates structural information, an order of magnitude more photons are absorbed and promptly deposit energy in the sample. This energy is promptly released by the emission of photoelectrons, extensive ionization, electronic rearrangement, structural rearrangement and ultimately, Coulomb explosion. That is, for hard X-rays the ratio of the elastic scattering cross section for an isolated atom to the sum of its inelastic scattering cross sections is small and hence unfavorable, particularly when compared with corresponding properties for electron scattering (see *e.g.* Henderson, 1995 ▸). However, the exact nature and time course of these damage processes and how these might vary from sample to sample are not yet known although these factors are significant for selecting the optimum duration of the X-ray pulse (is shorter always better?) and its wavelength. In a significant paper that appeared since submission of the review, Rudenko *et al.* (2017 ▸) point out that unexpectedly, the ionization of a molecule that contains a heavy atom such as iodine is considerably enhanced over that associated with an individual iodine atom. In their experiments, the extent of primary radiation damage depends on the electronic and molecular environment of the absorbing atom. Heavy atoms are of course critical to phasing by anomalous scattering, and their oxidation state and detailed stereochemistry within the complex metal centers at the active site of, for example, photosystem II are essential to overall function. There are indications that the Fe atoms in the two chemically identical 4Fe – 4S clusters in ferredoxin from *Clostridium* differ in their extent of radiation damage from XFEL illumination (Nass *et al.*, 2015 ▸). The molecules CH_3_I and C_6_H_5_I studied by Rudenko *et al.* (2017 ▸) are very small by biological standards but it seems entirely likely that their general result will hold for heavy atoms in biological macromolecules: atoms that are chemically identical may not be identical in their propensity for radiation damage! Clearly, more work is needed to establish the extent and time course of primary radiation damage in proteins, complicated by the likelihood that these may be unique to every atom.

A second favorite is the development of new approaches to the accurate extraction of structure amplitudes from the large array of partial scattering intensities, each from a separate microcrystal. All XFEL diffraction patterns are Laue patterns since the crystal is effectively stationary during the X-ray pulse. However, the bandpass of the pulse is sufficiently narrow that all spots are partial, particularly at lower resolution. This contrasts with the much wider bandpass typical at storage rings, where no spots are partial – all are fully recorded – and accurate structure amplitudes can readily be extracted (Ren & Moffat, 1995 ▸). Extraction of structure amplitudes by brute force Monte Carlo methods does work, in essence by averaging a very large number of measurements each of which is highly inaccurate; but large quantities of precious sample are required. Methods that effectively model crystal disorder and lead to refinable estimates of partiality of each Laue spot are definitely called for.

This timely review by Spence will stimulate and provoke the practitioners in this rapidly developing field.
